# Racial and Ethnic (In)equity in Development of Power Through Place-Based Initiatives

**DOI:** 10.3390/healthcare12232486

**Published:** 2024-12-09

**Authors:** Mina Silberberg, Matthew E. Dupre, James Moody, Meera Patel, Anika Vemulapalli, Douglas Easterling

**Affiliations:** 1 Department of Family Medicine and Community Health, Duke University School of Medicine, Durham, NC 27710, USA; 2 Department of Population Health Sciences, Duke University School of Medicine, Durham, NC 27710, USA; matthew.dupre@duke.edu; 3 Department of Sociology, Duke University, Durham, NC 27710, USA; jmoody77@duke.edu; 4 Duke Margolis Institute for Health Policy, Duke University, Durham, NC 27710, USA; meera.patel@duke.edu (M.P.); anika.vemulapalli@duke.edu (A.V.); 5 Department of Social Sciences and Health Policy, Wake Forest University, Winston-Salem, NC 27109, USA; dveaster@wakehealth.edu

**Keywords:** race/ethnicity, health equity, population health, health politics, place-based initiatives

## Abstract

**Background**: Place-based initiatives (PBIs) invest in a geographic area and often build community power to improve well-being. However, there can be differences in results for different groups within a community. **Methods**: In six communities, we measured differences in “power to” by race/ethnicity at two points for the first phase of the PBI Healthy Places North Carolina (HPNC) using five indicators: (1) representation in network of actors collaborating to improve health, (2) leadership attributes, (3) perceived change in attributes due to HPNC, (4) network centrality, and (5) perceived change in network ties due to HPNC. **Results**: Latine populations were underrepresented. In four (majority White) communities, there were indications of White advantage. In one, White centrality was greater than non-White. In another, White actors consistently rated themselves higher for leadership attributes. In two, a gap in leadership attributes favoring White actors appeared at Wave 2. In two counties with African American majorities, non-White attributes ranked higher than White. **Conclusions**: Each indicator provided unique insight. Results provide new evidence of measurement validity and reliability. Results indicate that when PBIs designed to address the needs of low-resource communities do not proactively concern themselves with racial/ethnic equity and power (as HPNC would do in the years after this study), they may result in greater White benefit from PBI or failure to close existing gaps. Findings aligned with the “political reality” model of the correspondence between the size of African American population and their perceived self-efficacy. Changes over time and inter-county differences confirm need for early measurement of power differences and changes.

## 1. Introduction

### 1.1. Overview

Place-based initiatives (PBIs)—focusing investment and capacity-building on a contained geographic area—are one approach taken by public sector and philanthropic actors to address high rates of poverty, poor health, and other aspects of well-being [1]. While results are mixed, some evaluations of these initiatives indicate success in intermediary outcomes, such as increased “social capital” and leadership capacity [2,3], and longer-term outcomes, such as decreases in hospital readmission rates, obesity, and low birthweight [3,4,5].

However, new research has identified the potential for inequity in how the ultimate benefits of PBIs are distributed. This article makes a case for the need to assess the equity of changes in power throughout the initiative, delineates specific challenges associated with this type of evaluation, and describes a methodology that addresses those challenges. It then presents the results of using this methodology to assess racial/ethnic equity of power-building during the first phase of Healthy Places North Carolina (HPNC), a PBI. The Discussion and Conclusion address the implications of these results for the conduct and evaluation of PBI.

### 1.2. Place-Based Initiatives

PBIs vary in strategies and goals. What unites them and distinguishes them from other approaches is that they target a well-defined geographic area. Other common characteristics include a focus on low-resource communities, cross-sector collaboration toward a common goal, an emphasis on resident involvement, and a relatively long time frame [1]. The Comprehensive Community Initiatives (CCIs) model—community and system change efforts that address multiple issues of low-income neighborhoods while engaging community residents—has been the PBI approach most commonly used by foundations, at least up through 2010. More recent PBIs have used models in which local strategy development and implementation are more organic and fluid [6,7]. In the health field, some observers are calling for even greater use of PBIs, given the heightened attention to the health effects of local built and socioeconomic environments [8].

### 1.3. The Need to Assess Intra-Community Equity in PBIs

Early PBIs implemented in the 1990s aspired to improve health on a community-wide level, but more recent PBIs have focused more specifically on reducing racial, ethnic, and economic disparities in health. This is not accidental. Inequitable investment in different geographic communities (majority Black vs. majority White, rural vs. urban) is one source of health inequity (defined by the World Health Organization as “systematic differences in the health status of different population groups”) [9] and one of the arguments for a greater focus on PBIs [8].

While PBIs have pursued equity by focusing investments on low-resource communities, there has not always been explicit attention to the potential for disparate impact of PBI on subgroups within the communities involved. Even in the last few years, with rising attention to the role of social conditions in shaping health at a given moment [8], there has been very little recognition of the ways in which those social conditions can lead to different intervention results for different subpopulations. However, a small body of literature has begun to explore inequitable outcomes within communities participating in PBIs. For example, a recent study indicated that youth who lived in counties experiencing greater place-based investment during their adolescence are likely to go on to higher education and income levels than those who experienced less place-based investment. However, deeper inquiry showed that all the benefit was derived by those who migrated outside of the counties in which they had lived as teenagers; in other words, the counties receiving significant place-based investment were losing the residents who most benefited from it—what is often called a “brain drain” [10]. Another study by the same team showed that PBI in formerly redlined areas was associated with increased property values. However, it was also associated with reductions in the share of Black homeowners in these areas [11]. Similarly, a study of the impact of vacant housing demolition indicated that the resulting gentrification had an adverse impact on working class residents and long-term renters’ sense of social exclusion and mental health [12]. PBIs clearly have the potential for inequitable outcomes.

### 1.4. The Need to Learn by Doing

If the needs of low-resource communities are the driving force behind most PBIs, then evaluation of PBIs must be attentive to the potential for inequitable outcomes within communities, and evaluation must be undertaken early to allow for course correction in case of early signs of inequitable outcomes. In fact, ongoing evaluation is essential to PBIs more generally. Based on a review of the literature on PBIs, Harder+Company [13] concluded that this work is intrinsically complex and adaptive, requiring “learning by doing” to be successful. Observers note that PBI strategy is and must be “emergent”; i.e., it must evolve in response to local context and changes in that context, results to date, and lessons learned along the way, including through ongoing evaluation [14].

### 1.5. Evaluating Differences and Changes in “Power to”

Ongoing evaluation of PBIs and learning by doing require assessment of early outcomes and of intermediary goals intended to bring about the initiatives’ ultimate results. A critical intermediary goal of most PBIs is to support communities in building the attitudes, knowledge, skills, experience, relationships, and infrastructure necessary to effectively identify and address their needs [2,3,13]. This can also be understood as building collective “power to” act effectively on the community’s behalf (vs. “power over” or domination of others) [15]. Inequities in building “power to” through PBIs and/or a failure to reduce existing inequities in power set the stage for continued or even widening inequities in health and therefore should be evaluated as PBI strategies emerge. In recent years, as concern for equity has become more central to conversations around PBI, so has a related concern about power: the power of investors relative to the power of communities and power within communities [7].

### 1.6. Challenges in Evaluating Power in PBIs

How can the impact of a PBI on changes in “power to” be evaluated? The very characteristics that necessitate ongoing evaluation of PBIs and intermediary outcomes—their ambitious goals, long-term nature, multi-faceted strategies, breadth, and complexity—also make it challenging [16,17]. One particular concern is the question of who is to be studied—or, for the purposes of this paper—whose power is to be assessed. A traditional evaluation assesses the actions of a specific set of implementers to affect the well-being of a specific set of beneficiaries. In that context, we can collect implementation data on the implementers and outcome data on the beneficiaries. In contrast, by activating key actors in a community, many PBIs aim to affect the behaviors of a larger pool of actors to bring about change for the entire community. Moreover, when we consider equity as a goal for PBIs, we must be as concerned with who is not affected by these initiatives as who is. Immediately, our group of “evaluation subjects” has broadened to include people whom the initiative has touched only indirectly and people who have not been touched at all—an omission with consequences.

Surveying only those who were working directly with the funder would create a self-fulfilling prophecy; we would likely see important actors making changes in their networks because we had by default defined important actors as those working with the funder. Moreover, we would be unable to assess whether the PBI was having the anticipated ripple effects on actors throughout the community. In order to address this problem, the survey sample could theoretically be expanded to encompass the residents and workforce of an entire county, since all of these might have something to contribute to a health improvement initiative. However, that would not be manageable; moreover, a PBI could not be expected to catalyze new relationships among all members of the community (nor would such an approach even be desirable, as networks grow to a point of diminishing efficiency). The challenge then is to develop a sample that does not merely replicate funder contacts but is meaningful in describing the landscape of those working or poised to work on community health and well-being.

### 1.7. Our Approach

In this paper, we describe an approach to evaluating changes in “power to” that was designed to address the needs delineated above—recognition of the intended community-wide scale of PBIs and use of an equity lens. We then look at the application of this approach to evaluation of differences in change in “power to” by race and ethnicity during the first six years of one place-based initiative (Healthy Places North Carolina, or HPNC). Racial and ethnic differences in health are among the most intractable inequities faced by our country [18]. A previous publication includes one-time findings on two “power to” metrics for four HPNC communities overall and disaggregated by sector, i.e., health, education, etc., not race and ethnicity [19]. This paper builds upon that earlier work by providing a more complete description of the methodology used and the reasoning behind it, looking at results by race/ethnicity, providing data on five metrics of “power to”, describing results for six communities, presenting two waves of data, and exploring HPNC’s impact on the distribution of power.

### 1.8. Study Context

Launched in May 2012, Healthy Places North Carolina (HPNC) was a USD 100 million PBI of the Kate B. Reynolds Charitable Trust designed to improve health outcomes in some of the most rural and socioeconomically disadvantaged communities in North Carolina. The approach of HPNC—described with additional information elsewhere [19,20,21,22]—was built on previous place-based initiatives (e.g., involving local residents, building capacity), while emphasizing the activation of actors outside the health sector and encouraging upstream efforts that were emergent and iterative within the community, rather than determined by a funder or predefined strategy. Many of those who had been engaged in place-based initiatives over the prior three decades believed that those initiatives had been hampered by the way that funders entered into communities. They called for funders to pay more attention and respect to local structures and politics and to support local actors in developing their own solutions [20].

HPNC was designed with this call in mind. Unlike the CCI model, HPNC is a cultivation-oriented PBI, in which the funder looks for and supports a variety of complementary efforts that have been developed in different segments of the community—with staff from the foundation (or government agency) actively exploring, activating, and cultivating new ideas. HPNC did not begin by convening local stakeholders to conduct a planning process. Instead, they relied on the Trust’s program officers and a range of partner organizations to identify key actors and potential actors for health improvement, build their capacity, and cultivate new ways of thinking and new types of work. While this approach distinguishes HPNC from many other PBIs, it does not change the importance of thinking about inequity; arguably, its emergent and community-driven nature only increases the importance of early evaluation and development of community “power to”.

Central to the health improvement paradigm for HPNC, and place-based approaches in general, are the local actors whom these initiatives are designed to empower and mobilize. These actors are the agents of change in the community and are vital to the effectiveness of place-based interventions. In HPNC’s emergent and community-driven approach, the goal was to engage local actors to (i) take greater initiative; (ii) think more strategically and in ways that may go against convention; (iii) connect and collaborate across sectoral and ideological boundaries; and (iv) learn from their experiences to increasingly deepen, broaden, and innovate their efforts to improve health in their community. At the outset of the initiative, the funder did not specify more precisely which actors it was seeking to affect, but rather was focused on the goal of stimulating new and more impactful work than previously existed.

To help achieve these goals, each HPNC county was assigned a program officer (PO) from outside the county who—in addition to carrying out traditional grant-making activities across a wide region of the state—worked in the community and met with individuals to gain an in-depth understanding of local actors, organizations, and issues in the county. The PO did not impose goals or strategies on community members. Rather, the PO served as a “cultivator” to help individuals and their organizations think about how to turn new ideas into actionable strategies, suggest potential collaborators with complementary goals, help identify capacity-building needs (and ways to address them), and suggest grant opportunities. The PO’s efforts were supplemented by locally based “program officer extenders” and by state, regional, and national partner organizations that provided capacity-building and technical assistance (e.g., Center for Creative Leadership, KaBOOM! Conservation Fund). The expectation was that this place-based attention and support would increase the capacity and interconnectedness of local actors to identify and solve health problems in their county.

In addition to building local capacity and local networks, the Trust provided over USD 100 million in grant support to the Healthy Places counties over the course of a decade. Sample grant-funded programs include co-location of a federally qualified health center and emergency department, a social care check-in program that gives Emergency Medical Services the ability to show up in a non-ambulatory vehicle to check on people at home before a situation escalates to a hospital visit, and a new resource center for individuals recovering from substance misuse. (For more details, see the Trust’s 10-year report [22].) In line with the cultivation approach—and in contrast to earlier PBIs—early grant-making was very much shaped by what the Trust staff and partners were learning from the communities about local residents’ concerns and opportunities for effective action. In later years, these priorities were used to focus grant-making in each county on a few key issues that could be addressed by targeted, complementary grant funds; the roots of these programmatic areas nonetheless lay in the counties’ own priorities and insights.

As has been described elsewhere [21,22], HPNC was carried out in two distinct phases. At the outset of HPNC, the funder was focused on the goal of increasing strategic action across the community. In line with other PBIs, the primary strategy to achieve equity was the inclusion in the initiative only of low-resourced counties, with the hope that this would lessen the gap in health and well-being between these counties and others in the state. There was an emphasis in both capacity-building and grant funding on broadening the types of actors involved in strategic action in these counties beyond the mainstream institutions (e.g., hospitals, health departments) that had dominated health-related efforts in the past. However, there was no proactive attention to the question of whether this either accentuated or attenuated racial or ethnic health disparities within the counties or disparities in who had the power to make change happen. At that point in time (2012), health foundations were only just beginning to orient their initiatives toward reducing racial and ethnic disparities in power; innovation was occurring primarily in the area of focusing on social determinants as a means of affecting population health.

The Trust’s orientation changed during the second phase of HPNC when it brought an explicit equity frame to the initiative. This resulted from changes in leadership at the Trust, as well as the growing focus on equity and power within the philanthropic field more generally. Along with this shift in focus, the Trust revised the model for HPNC to focus on building the power of traditionally disenfranchised residents while also elevating the importance of systems change. This ultimately led to strategic shifts in how local stakeholders were supported, as well as the types of groups that were supported.

This paper presents evaluation findings that reflect the first phase of HPNC. From 2013 to 2018, a team from Duke University was contracted to assess the impact of HPNC on intermediary outcomes. The primary goal of the evaluation was to assess changes across the HPNC communities in leadership attributes, the networks of actors working to improve health and well-being, organizations working in the communities, the kind of work being performed, and the communities overall. A secondary aim was to assess whether changes in leadership attributes and networks were equitable for different subpopulations within the communities.

During the evaluation time period, six communities were part of HPNC. Four had been brought into HPNC in the period from 2012–2013: Communities A, C, D, and F. (We have anonymized the communities out of respect for them. We use the terminology of “communities” because, although five of these are single counties, one is a two-county region that shares a common metropolitan area). Communities B and E were added in 2015. Although these six communities share a nexus of common disadvantages—high rates of poverty, unemployment, and depressed economies—they have distinct local histories, geography, racial/ethnic make-up, and sociopolitical culture that shape their community and the health issues they face.

The regional location and racial/ethnic demographics of these communities during the time of this evaluation are provided in Table 1. Region is specified for two reasons. First, program officers were assigned to specific regions, and there were some differences in implementation of the initiative by program officer [22]. However, our ability to describe consistent and distinct approaches to the work by region is limited by program officer turnover. Second, and more importantly, there were some common characteristics within regions. Of particular interest for this study, the non-White population in the western communities was very small, while in the central region, two of the three communities were majority African American/Black.

## 2. Materials and Methods

### 2.1. Design

The data presented here represent results of a survey that constituted one component of a larger set of evaluation activities undertaken by the Duke team. The survey was designed to capture characteristics of community leaders and relationships of cooperation and collaboration among them (or networks). It also solicited information on the contributions of HPNC to changes to both leaders and network relationships. The survey was a web-based questionnaire (programmed in Qualtrics; Qualtrics, Provo, UT, USA) that required approximately 20 min to complete. It was distributed in two waves in each community—once early on, but when HPNC had had a chance to have an initial impact; and once approximately two years later.

Because the evaluation was commissioned when Communities A, C, and F had already been part of the initiative for one year, they were first surveyed approximately two years from start. All other communities were initially surveyed approximately six months from start. The two waves of data collection were May to October of 2014 and June 2016 to January 2017 in the first four communities and June 2016 to January 2017 and November 2017 to June 2018 in the other two. We attempted to reach non-respondents by phone three times and offered to administer survey that way; two fluent Spanish speakers were available for phone administration.

### 2.2. Sample

Our challenge, as described in the Introduction, was to develop a manageable sampling frame that did not merely reflect those with whom the Trust had worked but provided a map of the key people involved in efforts to improve health and well-being in the county. Because our goal was to develop this complete map, we did not conduct a power analysis. Rather, we used purposive sampling to identify local actors who were deemed important (or potentially important) in efforts to improve their county’s health and drivers of health. For each community, we generated lists of individuals from four primary sources. First, the Trust program officers provided lists of individuals from their communities who represented key actors with whom they were already working or whom they thought could be important to their efforts (e.g., local officials, organizational leaders, community organizers).

Second, the evaluation team used information collected from environmental scans in each county to supplement and refine the lists provided by the program officers. Environmental scans consisted of review of local newspapers to understand major community concerns, trends, and events, and review of data from the U.S. Census [23], the NC Department of Commerce [24], the NC Department of Public Instruction [25], individual county community health assessments, and County Health Rankings and Roadmaps [26]. Based on this information, we used contacts in the communities and internet searches to identify potential respondents from geographic, sectoral, and racial/ethnic communities underrepresented in program officers’ lists.

Third, as part of the larger HPNC evaluation, we conducted key participant interviews (KPIs) with a subset of the lists provided by the program officers; at the end of each interview, respondents provided names of important actors in the county. We added these names to the survey lists. Fourth, when completing the survey, respondents were given the opportunity to name up to 10 additional individuals with whom they had relevant ties but who were not already on the list. (Nearly 70% of respondents provided no additional names, and about 90% of those who provided names listed 3 or fewer.) These individuals were added to the survey sample on a rolling basis.

For the second wave survey, we began with our initial survey sample from Wave 1, added all KPI respondents from both waves of data collection, and added all of the individuals named by initial respondents in Wave 1 who then responded to the survey or were mentioned by others more than twice.

### 2.3. Measures

Race and ethnicity and the categories defined for them are social constructs. In the contemporary United States, the term ethnicity is generally used to differentiate between people of Hispanic or Latine descent (i.e., with ancestry from Latin America (with some debate about ancestry from Spain and Portugal)) or not. Race and ethnicity are therefore not mutually exclusive, e.g., one can be Hispanic and White or Hispanic and Black. All approaches to measurement of race and ethnicity have advantages and disadvantages; we employed a commonly used practice of asking one question (“What is your race/ethnicity?”) and instructing respondents to choose as many as apply; this approach gives respondents maximum flexibility [27].

Response options for race/ethnicity were African American/Black, White, Hispanic/Latino, Native American/American Indian, Asian/Pacific Islander, and Other (with the opportunity to explain using free text). Few respondents chose more than one response. Therefore, for descriptive statistics, those choosing any of the first three response options were recoded as Non-Hispanic African American/Black, Non-Hispanic White, and Hispanic/Latino. Cell sizes for groups other than White and African American/Black were small. Therefore, for purposes of comparison of leadership attributes and network centrality, respondents were coded as White and Non-White.

“Power to” is a function of capacity, resources, and opportunities [15]. For our evaluation, we looked at three different dimensions of “power to”, with two dimensions associated with two metrics each, for a total of five indicators. The first dimension was representation of different racial/ethnic groups in the set of key actors resulting from survey administration. We did not assume that actors who did not come to our attention were uninvolved in attempting to promote health and well-being. However, we did assume that those who were less visible in this sphere were less likely to be identified through our sampling methods and therefore less likely to be among our respondents; lower visibility at a minimum would mean less potential to build on the capacity, resources, and opportunities associated with collaboration.

The second dimension of “power to” that we assessed was leadership attributes, a form of capacity. Survey participants were asked 15 questions about leadership attributes—the extent of aspects of leadership knowledge, identity, ability, and behavior. Responses were scored on a 5-point Likert scale (from 1 = “not at all” to 5 = “a great deal”). We worked with HPNC leadership to identify key domains relevant to their leadership building work and reviewed the relevant literature. We were able to adapt four items from existing surveys [28,29,30] and developed the remainder ourselves.

We pilot-tested all survey questions with individuals in our county who had similar roles to those of the intended respondents. We reviewed their responses and asked them what they understood the questions to mean and whether they were confused at all or had concerns about the wording. We then made small revisions accordingly. No further validity or reliability testing was performed at this point. For each of the items, the follow-up question “How has this changed because of HPNC?” was asked, with response options of increased, stayed the same, or decreased. For leadership attributes, therefore, we were able to look at changes in two indicators over time: leadership attributes and respondents’ perceptions that their leadership attributes were improving as a result of HPNC. Table 2 provides examples of the leadership attribute questions. Inter-item reliability testing for multi-item domains produced an overall Cronbach’s alpha of 0.77, indicating good performance. Measurement reliability was also indicated by similar patterns of high, moderate, and low scores by item across different counties and different time points [19], Supplemental Materials.

The third dimension of “power to” that we assessed was “network centrality”, a product of social network analysis (SNA). SNA is a means of examining social structures—like our map of key actors. It allows you to answer questions such as “how big is the network?”, “how well-connected to each other are the members of the network?”, and “who is especially well-connected to others?” These questions can be answered by looking at visual images or maps of the networks and also by statistics that are generated to measure connectedness. The maps show “nodes” (or network members) in the form of dots or something similar and ties between them as lines connecting the nodes. SNA provides a general tool for understanding the systems of complex relations that underlie community health; its use in our work provides a formal approach to addressing such questions, which have usually been tackled qualitatively.

To allow for SNA, our survey gathered detailed information on the number and type of connections among actors in the county. Respondents were asked to select all individuals whom they knew from a list of the others in their community’s starting sample and, as noted, to list up to 10 additional people with whom they connected to address community health and well-being. For each person who was selected or named, respondents were asked to indicate whether they had done the following activities together (yes or no): (1) “attended meetings, workshops, or trainings”; (2) “shared ideas, strategies, and/or planned activities”; (3) “worked together on grants and/or initiatives”; or (4) none of these activities. If they responded yes to any of these three, we considered them to be working with this person to address health and well-being.

Based on these data, we created a network map in which the “nodes” were individuals, and ties between them indicated that they were working together to address health and well-being. For each working relationship, we asked whether HPNC had helped to initiate or strengthen the relationship—yes or no—and assigned that value to the tie connecting the two people.

These data allowed us to measure our third dimension of “power to”—closeness centrality. Closeness centrality is a measure of how close each person in the network is to all the others (i.e., the inverse of the average number of nodes between that person and each of the others). Greater closeness means greater ease of reaching other nodes. It indicates how well-positioned a person is to communicate with others, influence others, and collaborate with others. In addition, when two nodes are not directly connected, those connected to each member of the pair have the option of leveraging that position to bring the others together or not, thereby potentially playing a gatekeeping role. Gatekeeping is another source of power for the well-connected; conversely, the further apart one node is from others—the more connectors s/he relies on—the less power s/he has [31]. As with leadership attributes, we were able to look at changes over time in two indicators associated with this dimension: closeness centrality and respondents’ perceptions that HPNC was growing their ties to others.

### 2.4. Analysis

We did not expect exact representation of county racial/ethnic composition in the network samples, and even small differences can be statistically significant. We therefore set a very conservative threshold for underrepresentation; a population was identified as being underrepresented only when its percentage in county make-up was more than 50% higher than its percentage in survey make-up.

Responses to attribute questions were generally skewed toward higher responses. For analytic purposes, therefore, responses were recoded as high (5 on Likert Scale) vs. not high, and differences by race/ethnicity for each question were tested using Chi2 and Fisher’s Exact Test, with a threshold for statistical significance of *p* < 0.05%; these tests allowed us to assess the probability that differences in the distribution of high responses by race/ethnicity were random or the result of an underlying association. Fisher’s is appropriate for smaller sample sizes.

Differences in race/ethnicity for proportion of respondents attributing improvement in a relationship to HPNC were tested using the same approach. Racial/ethnic differences in these variables were only designated when there was a clear pattern of significant difference in the same direction across a majority of leadership dimensions or of attributions of change to HPNC.

Differences in closeness centrality across subpopulations were analyzed using 95% confidence intervals. Iacobucci and colleagues have noted that, while SNA is commonly used for descriptive purposes, inferential analyses of difference, such as those performed here, are a powerful way of strengthening our confidence in patterns that visual SNA and descriptive statistics seem to reveal. They write:

Descriptive statistics on networks are certainly very informative, yet even more information may be obtained via inferential methods with statistical methods that allow testing hypotheses about network structure. For example, social network analysts might pose questions such as whether a set of actors are significantly different from others, to help formulate conclusions about patterns that appear different, making those assessments with greater confidence [32].

We conducted descriptive and bivariate analyses using Stata version 14.0 (StataCorp LP, College Station, TX, USA). We conducted network analyses with SAS version 9.4 (SAS Institute, Cary, NC, USA; data cleaning and scoring), PAJEK (Program for Analysis and Visualization of Very Large Networks; illustrations), and Adobe Illustrator version 15.1.0 (Adobe Systems Inc., San Jose, CA, USA). The study protocol was approved by the Duke University Health System Institutional Review Board.

## 3. Results

### 3.1. Overall Results

Response rates for the six communities and the two waves of data collection (measured as the percentage of individuals from the starting sample who completed the survey) ranged from 67% to 87.3%. Total sample sizes for the 12 instances of data collection are provided in Table 3.

Across the 12 administrations of the survey (6 communities × 2 time points), a majority of respondents were female; a majority were aged 46–65; and a majority had lived in the county in question for more than 10 years. As will be discussed in further detail, racial/ethnic composition of survey respondents was similar to that in the counties, with some important exceptions.

Overall, most respondents scored high in terms of awareness and attitudes, such as awareness of health issues, sense of agency, attitude toward collaboration, and whether they considered themselves a leader in the community. More strategic skill sets, such as connecting diverse individuals and organizations and using resources in new ways, received lower scores; ability to address significant challenges, e.g., identifying individual or organizational barriers, received lower scores yet.

In almost every community and every attribute, scores increased from Wave 1 to Wave 2 for each community; moreover, at both waves, large numbers of respondents attributed strengthening of these attributes to Healthy Places NC. Networks among actors in the individual communities were generally dense (i.e., there were many working relationships). Across communities, the number of ties grew substantially from Wave 1 to Wave 2, and at both time points, large numbers of respondents stated that individual relationships had been initiated or strengthened by HPNC. (For more on these findings, see [19].)

### 3.2. Differences by Race and Ethnicity

Network Representation. There is a common element across all six communities of underrepresentation of the Latine/Hispanic population in the networks—with representation ranging from 0 to 2.5 percent. This was true in both the first and second waves of data collection. Burke County was unusual among the six communities in having a measurable Asian population (3.5%) and this group, like the Latine/Hispanic population, was underrepresented in Community B’s network at both time points. At Wave 1, we also found underrepresentation of the Black/African American population in Community F, which was approximately around one-quarter Black/African American at the time. At Wave 2, this finding had disappeared.

Leadership Attributes. Table 4 provides results of leadership attribute and network centrality assessment. In Community B (western region), White actors had higher self-reported leadership attributes than non-White actors at both waves of data collection; while non-White actors were more likely to cite enhancement of leadership attributes through HPNC at Wave 1, that pattern had reversed itself at Wave 2.

In two communities—A in the west and D in the central region—there was no difference in leadership attributes by race/ethnicity at Wave 1, but at Wave 2, White actors had higher-rated leadership attributes than non-White. In Community D, furthermore, White actors were more likely than non-White actors to cite enhancement of leadership attributes resulting from HPNC.

In a fourth community—F, in the eastern part of the state—there was no difference in self-reported leadership attributes at either time point. While non-White actors were more likely than White actors to report strengthening of leadership attributes due to HPNC and there was some indication of non-White actors having higher leadership attributes than Whites at Wave 2, the pattern did not meet our threshold for difference.

In two communities, C and E, non-White actors had higher scores on self-reported leadership attributes than did White actors at both time points and were more likely than White actors to attribute improvements in leadership attributes to HPNC. Both C and E, located in the state’s central region, were also the only majority Black/African American communities studied.

### 3.3. Network Centrality

In five of the six communities, there was no difference in network centrality by race/ethnicity at either point in time and no difference in attribution of enhancement of relationships to HPNC. However, in Community F, White actors in the network had higher network centrality scores than non-White actors at both points in time (CI of 95%).

## 4. Discussion

### 4.1. Interpretation of Key Findngs

In this evaluation of the differential impact of a PBI on racial/ethnic power to improve the health of their communities, we utilized five indicators: (1) representation in the network of actors collaborating to improve health, (2) leadership attributes, (3) change in attributes due to HPNC, (4) network centrality, and (5) network relationships begun or enhanced due to HPNC. While this was not a formal assessment of our metrics, a number of findings provide some additional evidence of validity and reliability beyond what was previously reported.

- The Latine population is consistently underrepresented in the communities’ collaborative networks. This finding conforms to expectations. At the time of the project, most of the Latine population in North Carolina was foreign-born (nearly two-thirds), and half spoke English less than very well [33]. Moreover, during the time period covered by this evaluation, none of HPNC’s key staff spoke Spanish—a circumstance that was later changed.
- Some differences across communities represent logical patterns. For example, in the two majority Black/African American communities, non-White actors had higher scores on self-reported leadership attributes than did White actors. This aligns with empirical research and the “political reality model” found in political science, which indicates that African Americans are more likely to see themselves as politically efficacious when they live in majority-Black communities and have been able to effect change [34]. Conversely, the four communities where there was indication of White advantage in self-perceived leadership attributes or network centrality were the four majority-White communities (71.5% to 93% White). It should also be noted, however, that this finding begs the question of whether the leadership attribute metric reflects individual leadership ability or collective power. The overall improvement in attributes and the large number of respondents attributing change in these leadership attributes to HPNC suggests that at least part of what drives responses is perceived individual ability. Nonetheless, the meaning of this metric and its association with county composition deserve more research; formal cognitive interviewing on these leadership attribute questions would be valuable for exploring how experiences of individual and collective power contribute to responses overall and the ways in which responses vary by county composition.
- Within counties, measures were usually consistent across the two time points, suggesting measurement reliability. At the same time, the limited cases of longitudinal change in distribution of leadership attributes were usually internally consistent with patterns of reported change in leadership attributes as a result of HPNC.

It is also clear that the metrics used in this study are not all measuring the same thing. Logically, the variable of underrepresentation in the network is distinct from the other two variables, which measure qualities of those who are in the network and are not dependent on the extent of concordance between community and network representation. More surprisingly, leadership attributes and network centrality did not paint the same picture, with leadership attributes indicating racial/ethnic differences in all counties and network centrality indicating difference only in Community F. Moreover, in Community F, leadership attributes and network centrality painted opposing pictures. Whether these findings indicate a problem with construct validity or rather logical distinctions between different dimensions of “power to” is a subject for further study.

Since our measures require further validation, we must be careful about over-interpreting what this study tells us about power dynamics in the communities studied during the first phase of HPNC. As noted above, our findings indicate that our measures were picking up some meaningful signals, but the exact meaning of those signals and their precision are unknown. However, findings in Communities A, B, D, and F—the four majority-White communities—suggest the potential for PBIs that have a concern for low-resource communities but do not pay explicit attention to racial/ethnic equity and issues of power to reinforce or even widen historical patterns of exclusion and disempowerment for non-White communities in the United States. Indeed, it was concern for this possibility that led to HPNC’s later shift to explicitly address both racial/ethnic equity and power [21,22].

The findings indicating reinforcement and widening of power inequity lend support to the contention that inequities in intermediary outcomes related to “power to” should be evaluated as PBIs are being implemented. At the same time, differences among the findings indicate the importance of not making assumptions about the impact of PBIs and the importance of understanding how county context may affect power-building. In particular, they indicate the value of studying power in the county at baseline and being attuned to differences in county dynamics associated with racial composition.

### 4.2. Qualifications and Limitations

It is important to emphasize that the findings reported here do not reflect the ultimate effect of HPNC on local power dynamics. As noted above, this evaluation was carried out during the first 5 years of HPNC, prior to a shift to an explicit focus on building the power of grassroots groups and people of color; we do not know how our measures would have performed in this later period.

Moreover, we cannot demonstrate a definitive correspondence between these patterns and health outcomes. Assessing the impact of PBI on health outcomes is complicated by a number of factors, including the long time frames that are generally necessary for change to take place through PBIs and the impact of external forces [35]. This was certainly the case during the life of HPNC, with COVID being particularly disruptive to health and community socioeconomic conditions. Studies that are able to employ longer time frames should incorporate assessment of the association between patterns of change in “power to” and who most benefits from the ultimate outcomes of PBIs.

Another limitation of this study is the fact that leadership attributes are self-reported (although this is common). Finally, this study is limited in that the web-based survey was programmed in English. However, follow-up calls were made to all of those who did not respond to the web-based survey, with two fluent Spanish speakers available for phone administration if it should be needed.

Despite these limitations, this paper adds to the literature, indicating the importance of assessing the equity of PBI impacts and bringing to the fore the importance of early assessment of the intermediary outcome of “power to”. It also suggests some approaches to sampling and measurement of power that proved to warrant further attention.

## 5. Conclusions

Each of the indicators utilized in this paper provided different insights into the capacity of racial and ethnic subgroups to act on behalf of their communities and the changes in that capacity over the course of and as a result of a PBI. Results also add to evidence of measurement validity and reliability. Further research is needed on the methodology utilized here to develop a sample of community actors for survey purposes, including qualitative research on the alignment between the resulting samples and the perspectives of key community actors and observers on who constitute key players. We also recommend validity testing in other contexts of the metrics shared here, as well as development and testing of alternative metrics.

Substantively, our findings suggest the possibility—in some contexts—of greater White benefit for power-building from PBIs or at least failure to close existing gaps when the sponsor is not explicitly paying attention to racial/ethnic disparities in power. Findings align with the “political reality” model of the correspondence between size of African American population and perceived self-efficacy. Long-term studies are needed that allow us to explore the association between patterns of change in “power to” and who most benefits from PBIs. However, the changes over time and inter-county differences found here provide evidence of the need for early measurement, including at baseline, to understand the specific county context and reinforce the need for sponsors and implementers of PBIs to commission ongoing evaluation of these initiatives and assess equity of PBI effects, revising strategy as needed to achieve more equitable outcomes. They also support the value of bringing an equity and power lens to PBIs, as would later be performed for HPNC.

## Figures and Tables

**Table 1:** Community Racial and Ethnic Make-up, Clustered by Region.

	Western NC Region		Central NC Region			Eastern NC Region
Racial/Ethnic Groups	Community A #	Community B ~	Community C #	Community D #	Community E ~	Community F #
Black/African American	4.2%	6.5%	53.1%	19.0%	56.8%	26%
White	93.0%	83.0%	40.7%	77.9%	37.2%	71.5%
Latine/Hispanic	5.5%	5.1%	2.3%	6.1%	3.9%	7.0%
Native American/American Indian	0.7%	---	3.8%	---	---	0.9
Asian	----	3.5%	---	---	---	---

Legend: # = Original four communities. ~ = Communities added in 2015. Data Source: US Census Bureau [23].

**Table 2:** Sample Leadership Survey Questions.

Domain	Sample Question
Understanding of health and health improvement	To what extent have you identified new ways to improve the health and well-being of your county?
Identities and responsibilities	To what extent do you see your professional or volunteer work as contributing to the health and well-being of your county?
Ability to leverage differences among groups of people/Ability to strategically assess networks and stakeholders	To what extent are you able to identify individuals and/or organizations that can help improve the health and well-being of your county?
Ability to lead through change	To what extent are you able to effectively work with others during periods of uncertainty and change?
Ability to manage energy	To what extent do you recruit others to join you in your efforts to improve your county’s health and well-being?
Self-awareness	To what extent do you understand your skills and abilities as a leader?
Agency	To what extent are you able to take action to improve the health and well-being of your county?
Attitude toward collaboration	To what extent do you believe that collaboration can help improve the health and well-being of your county?

**Table 3:** Sample Size *.

	Wave 1	Wave 2
Community Al #	87	75
Community B ~	107	100
Community C #	92	75
Community D #	80	113
Community E ~	106	73
Community F #	92	97

* Includes respondents from starting sample and added-name respondents. Legend: # = original communities, with Wave 1 survey data collected in May–September 2014 and Wave 2 from June 2016–January 2017. ~ = added communities, with Wave 1 survey data collected March 2015–August 2015 and Wave 2 from November 2017–June 2018.

**Table 4:** Racial/Ethnic Differences in Leadership Attributes and Network Centrality in HPNC Counties.

Location	Overall Racial/ Ethnic Make-up	Leadership Attributes ^a^			Network Centrality ^b^		
		Wave 1 Leadership Attributes	Wave 2 Leadership Attributes	Changes in Attributes Due to HPNC	Network Centrality, Wave 1	Network Centrality Wave 2	Relationships Begun or Enhanced by HPNC
WESTERN NORTH CAROLINA REGION							
Community A #	Majority White	Ø	W > NW	Time 1:Ø Time 2:Ø	Ø	Ø	Time 1: Ø Time 2: Ø
Community B ~	Majority White	W > NW	W > NW	Time 1: NW > W Time 2: W > NW	Ø	Ø	Time 1:Ø Time 2: Ø
CENTRAL NORTH CAROLINA REGION							
Community C #	Majority African American	NW > W	NW > W	Time 1: NW > W Time 2: NW > W	Ø	Ø	Time 1: Ø Time 2: Ø
Community D #	Majority White	Ø	W > NW	Time 1: W > NW Time 2: W > NW	Ø	Ø	Time 1: Ø Time 2: Ø
Community E ~	Majority African American	NW > W	NW > W	Time 1: NW > W Time 2: NW > W	Ø	Ø	Time 1: Ø Time 2: Ø
EASTERN NORTH CAROLINA REGION							
Community F #	Majority White	Ø	Ø	Time 1: NW > W Time 2: NW > W	W > NW	W > NW	Time 1: Ø Time 2: Ø

Legend: W = White. NW = Non-White. NW > W or W > NW indicates a statistically significant difference. Ø = No difference. # Wave 1 survey data were collected in May–September 2014 and Wave 2 from June 2016–January 2017. ~ Wave 1 survey data were collected March 2015–August 2015 and Wave 2 from November 2017–June 2018. ^a^ Leadership attribute question responses were recoded as high (5 on Likert Scale) vs. not high, and differences by race/ethnicity for each question were tested using Chi2 and Fisher’s Exact Test, with a threshold for statistical significance of *p* < 0.05%. Racial/ethnic differences for leadership attributes overall were designated when there was a clear pattern of significant difference in the same direction across a majority of leadership dimensions. Differences in race/ethnicity for proportion of respondents attributing improvement in a relationship to HPNC (yes/no) were tested using Chi2 and Fisher’s Exact Test, with a threshold for statistical significance of *p* < 0.05%. Racial/ethnic differences for change due to HPNC overall were designated when there was a clear pattern of significant difference in the same direction across a majority of the attribute change questions. ^b^ Differences in closeness centrality across subpopulations were analyzed using 95% confidence intervals.

## Data Availability

Data are available upon request.
